# 1,3-Bis(propan-2-yl)naphthalene

**DOI:** 10.1107/S1600536811047854

**Published:** 2011-11-16

**Authors:** Bernd Schröder, Ligia Rebelo Gomes, John Nicolson Low

**Affiliations:** a CICECO, Departamento de Química, Faculdade de Ciências, Universidade do Aveiro, 3810-193 Aveiro, Portugal; b CIAGEB-Faculdade de Ciências de Saúde, Escola Superior de Saúde da UFP, Universidade Fernando Pessoa, Rua Carlos da Maia, 296, P-4200-150 Porto, Portugal; c Department of Chemistry, University of Aberdeen, Meston Walk, Old Aberdeen, AB24 3UE, Scotland.

## Abstract

In the title compound, C_16_H_20_, one of the isopropyl groups shows almost equal displacements [1.252 (1) and −1.270 (1) Å] of its methyl-C atoms from the mean plane of the naphthalene ring system, while the other shows asymmetric displacements [1.586 (2) and −0.315 (1) Å]. In the crystal, the mol­ecules are linked into sheets lying in the *ab* plane by three C—H⋯π contacts, two involving donors belonging to the isopropyl groups and the third a donor atom from the naphthalene ring system. The different orientations of the isopropyl groups might be attributed to the fact that the C—H⋯π inter­action involving one of them is enhanced by the C—H⋯π inter­action involving the aromatic ring.

## Related literature

For background to diisopropyl­naphthalenes, see: Addison (1983 ▶); Brzozowski *et al.* (2001 ▶); Collin *et al.* (2003 ▶).
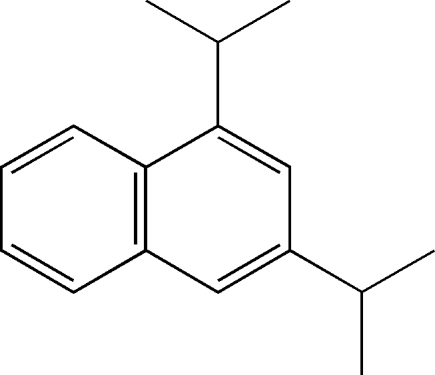


## Experimental

### 

#### Crystal data

C_16_H_20_
*M* *_r_* = 212.32
Orthorhombic, 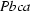

*a* = 16.1044 (12) Å
*b* = 8.2099 (5) Å
*c* = 19.0303 (13) Å
*V* = 2516.1 (3) Å^3^
*Z* = 8
Mo *K*α radiation
μ = 0.06 mm^−1^
*T* = 150 K
0.22 × 0.20 × 0.04 mm

#### Data collection

Bruker SMART APEX CCD diffractometer
Absorption correction: multi-scan (*SADABS*; Sheldrick, 2003 ▶) *T*_min_ = 0.986, *T*_max_ = 0.998
12847 measured reflections
2751 independent reflections
2240 reflections with *I* > 2σ(*I*)
*R*_int_ = 0.036

#### Refinement

*R*[*F* ^2^ > 2σ(*F* ^2^)] = 0.046
*wR*(*F* ^2^) = 0.108
*S* = 1.04
2751 reflections
145 parameters
H-atom parameters constrained
Δρ_max_ = 0.19 e Å^−3^
Δρ_min_ = −0.21 e Å^−3^

### 

Data collection: *APEX2* (Bruker, 2004 ▶); cell refinement: *SAINT* (Bruker, 2004 ▶); data reduction: *SAINT*; program(s) used to solve structure: *SHELXS97* (Sheldrick, 2008 ▶); program(s) used to refine structure: *SHELXL97* (Sheldrick, 2008 ▶); molecular graphics: *PLATON* (Spek, 2009 ▶); software used to prepare material for publication: *SHELXL97*.

## Supplementary Material

Crystal structure: contains datablock(s) global, I. DOI: 10.1107/S1600536811047854/hb6496sup1.cif

Structure factors: contains datablock(s) I. DOI: 10.1107/S1600536811047854/hb6496Isup2.hkl

Supplementary material file. DOI: 10.1107/S1600536811047854/hb6496Isup3.cml

Additional supplementary materials:  crystallographic information; 3D view; checkCIF report

## Figures and Tables

**Table 1:** Hydrogen-bond geometry (Å, °) *Cg*1 and *Cg*2 are the centroids of the C1–C4/C9/C10 and C5–C10 rings, respectively.

*D*—H⋯*A*	*D*—H	H⋯*A*	*D*⋯*A*	*D*—H⋯*A*
C8—H8⋯*Cg*2^i^	0.95	2.88	3.7399 (15)	151
C11—H11⋯*Cg*1^i^	1.00	2.98	3.8289 (13)	144
C31—H31⋯*Cg*2^ii^	1.00	2.68	3.6048 (14)	155

Symmetry codes: (i) 
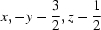
; (ii) 
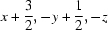
.

**Fig. 1.**
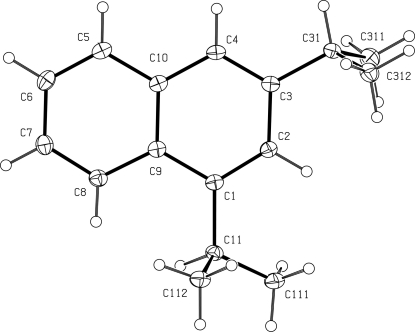
A view of (I) with displacement ellipsoids drawn at the 30% probability level.

**Fig. 2.**
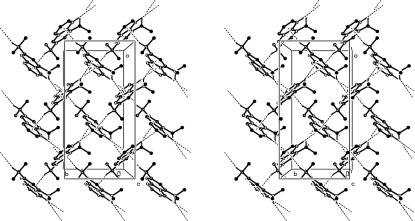
Stereoview of the sheet formed by C—H···π interactions. Hydrogen atoms not involved in the motifs are not included.

## supplementary crystallographic information

### Comment

Alkylated naphthalenes have a wide spectrum of applications, ranging from solvents, insulating material, heat transfer fluids, dye works auxiliary and specialty lubricants, (Collin *et al.*, 2003). Due to a variety of novel applications, diisopropylnaphthalene isomers have recently become of interest; they are used in the food packaging industry and as a plant growth regulator; furthermore, they have been introduced as PCB replacement fluids, (Addison,1983). The application of diisopropylnaphthalene isomer mixtures as solvents for carbonless copy paper, in its formulation known as KMC - Kureha Micro Capsule Oil - is of special importance. Some aromatic surfactants are partially based on propylated naphthalenes whose isomeric mixtures are used as water repelling agents for a variety of applications, such as corrosion protections, marine paints, resins,inks, coatings, plasticizers, or electrical, electronic and mechanical applications.

The main components of diisopropylnaphthalene isomer mixtures are 2,6-diisopropylnaphthalene and 2,7-diisopropylnaphthalene, contributing each with *ca* 40% to the mixture, while a few percent are made of the 1,3-, 1,6- and 1,7-diisopropylnaphthalenes, (Brzozowski *et al.*, 2001). Diisopropylnaphthalenes with isopropyl groups positioned in adjacent ring positions are usually not detected in the mixtures. Separating all isomers or synthesizing them in pure form remains a challenging task.

The 1,3-isomer (I) is shown in Figure 1. The isopropyl methyl groups are oriented *cis* with respect to atom C2. The naphthalene-isopropyl torsion angles show that the orientations of the isopropyl groups around the C1—C11 and C3—C31 bonds with respect to the naphthalene ring is different in each case, *e.g.* C2—C1—C11—C111/C112 - 23.24 (17)°/100.54 (14)° and C2—C3—C31—C311/C312 64.27 (16)°/-60.14 (15)°. The orientation of the isopropyl group attached to C1 is unexpected in that unlike the group attached to C3 in which the hydrogen atom attached to C31 lies in the plane of the plane of the naphthalene ring that attached to C11 does not do so.

These orientations position the hydrogen atoms attached to C11 and C31 so that the molecules are linked by three C–H···π contacts to form a sheet lying in the *ab* plane, Table 1, Figure 2.

### Experimental

Crystal of C_16_H_20_ were obtained from Rütgers Novares GmbH, Duisburg, Germany, with a stated purity greater than 98 percent and used as such.

### Refinement

Molecule (I) crystallized in the orthorhombic system; space group Pbca from the systematic absences. H atoms were treated as riding atoms with C—H(aromatic), 0.95 Å, C—H(aliphatic), 1.00 Å, with *U*_iso_ = 1.2Ueq(C), *C*—*H*(methyl), 0.98 Å,with *U*_iso_ = 1.5Ueq(C). The positions of the hydrogen atoms were confirmed in a final difference map.

### Crystal data

**Table:**

C_16_H_20_	*D*_x_ = 1.121 Mg m^−^^3^
*M**_r_* = 212.32	Mo *K*α radiation, λ = 0.71073 Å
Orthorhombic, *P**b**c**a*	Cell parameters from 421 reflections
*a* = 16.1044 (12) Å	θ = 3.9–23.6°
*b* = 8.2099 (5) Å	µ = 0.06 mm^−^^1^
*c* = 19.0303 (13) Å	*T* = 150 K
*V* = 2516.1 (3) Å^3^	Block, colourless
*Z* = 8	0.22 × 0.20 × 0.04 mm
*F*(000) = 928	

### Data collection

**Table:**

Bruker SMART APEX CCD diffractometer	2751 independent reflections
Radiation source: fine-focus sealed tube	2240 reflections with *I* > 2σ(*I*)
graphite	*R*_int_ = 0.036
ω scans	θ_max_ = 27.1°, θ_min_ = 2.5°
Absorption correction: multi-scan (*SADABS*; Sheldrick, 2003)	*h* = −12→20
*T*_min_ = 0.986, *T*_max_ = 0.998	*k* = −10→8
12847 measured reflections	*l* = −24→18

### Refinement

**Table:**

Refinement on *F*^2^	Primary atom site location: structure-invariant direct methods
Least-squares matrix: full	Secondary atom site location: difference Fourier map
*R*[*F*^2^ > 2σ(*F*^2^)] = 0.046	Hydrogen site location: inferred from neighbouring sites
*wR*(*F*^2^) = 0.108	H-atom parameters constrained
*S* = 1.04	*w* = 1/[σ^2^(*F*_o_^2^) + (0.0398*P*)^2^ + 0.9272*P*] where *P* = (*F*_o_^2^ + 2*F*_c_^2^)/3
2751 reflections	(Δ/σ)_max_ < 0.001
145 parameters	Δρ_max_ = 0.19 e Å^−^^3^
0 restraints	Δρ_min_ = −0.21 e Å^−^^3^

### Special details

**Table:**

Geometry. All e.s.d.'s (except the e.s.d. in the dihedral angle between two l.s. planes) are estimated using the full covariance matrix. The cell e.s.d.'s are taken into account individually in the estimation of e.s.d.'s in distances, angles and torsion angles; correlations between e.s.d.'s in cell parameters are only used when they are defined by crystal symmetry. An approximate (isotropic) treatment of cell e.s.d.'s is used for estimating e.s.d.'s involving l.s. planes.
Refinement. Refinement of *F*^2^ against ALL reflections. The weighted *R*-factor *wR* and goodness of fit *S* are based on *F*^2^, conventional *R*-factors *R* are based on *F*, with *F* set to zero for negative *F*^2^. The threshold expression of *F*^2^ > σ(*F*^2^) is used only for calculating *R*-factors(gt) *etc*. and is not relevant to the choice of reflections for refinement. *R*-factors based on *F*^2^ are statistically about twice as large as those based on *F*, and *R*- factors based on ALL data will be even larger.

### Fractional atomic coordinates and isotropic or equivalent isotropic displacement parameters (Å^2^)

**Table:**

	*x*	*y*	*z*	*U*_iso_*/*U*_eq_	
C1	0.35224 (7)	0.39257 (16)	0.18964 (7)	0.0211 (3)	
C11	0.30803 (7)	0.23796 (16)	0.16559 (7)	0.0230 (3)	
H11	0.2519	0.2366	0.1881	0.028*	
C111	0.29530 (9)	0.22710 (18)	0.08610 (7)	0.0321 (3)	
H11A	0.2666	0.1252	0.0746	0.048*	
H11B	0.3494	0.2295	0.0625	0.048*	
H11C	0.2617	0.3196	0.0702	0.048*	
C112	0.35537 (8)	0.08727 (17)	0.19147 (8)	0.0288 (3)	
H11D	0.3266	−0.0114	0.1758	0.043*	
H11E	0.3580	0.0885	0.2429	0.043*	
H11F	0.4118	0.0884	0.1722	0.043*	
C2	0.40246 (7)	0.47957 (16)	0.14496 (7)	0.0223 (3)	
H2	0.4066	0.4450	0.0974	0.027*	
C3	0.44854 (7)	0.61884 (15)	0.16637 (7)	0.0221 (3)	
C31	0.50394 (8)	0.70740 (16)	0.11426 (7)	0.0250 (3)	
H31	0.5322	0.7979	0.1400	0.030*	
C311	0.45361 (9)	0.78327 (18)	0.05444 (8)	0.0332 (3)	
H31A	0.4912	0.8394	0.0220	0.050*	
H31B	0.4138	0.8616	0.0738	0.050*	
H31C	0.4236	0.6975	0.0292	0.050*	
C312	0.57143 (8)	0.59413 (18)	0.08548 (8)	0.0314 (3)	
H31D	0.6061	0.6539	0.0519	0.047*	
H31E	0.5454	0.5013	0.0618	0.047*	
H31F	0.6060	0.5548	0.1243	0.047*	
C4	0.44313 (7)	0.66789 (16)	0.23492 (7)	0.0225 (3)	
H4	0.4738	0.7604	0.2500	0.027*	
C5	0.38868 (8)	0.63261 (16)	0.35543 (7)	0.0257 (3)	
H5	0.4211	0.7224	0.3708	0.031*	
C6	0.33889 (8)	0.55295 (18)	0.40250 (7)	0.0283 (3)	
H6	0.3372	0.5871	0.4502	0.034*	
C7	0.29021 (8)	0.42038 (17)	0.38021 (7)	0.0274 (3)	
H7	0.2546	0.3671	0.4127	0.033*	
C8	0.29370 (8)	0.36748 (17)	0.31199 (7)	0.0245 (3)	
H8	0.2609	0.2769	0.2981	0.029*	
C9	0.34551 (7)	0.44543 (16)	0.26157 (7)	0.0211 (3)	
C10	0.39261 (7)	0.58325 (16)	0.28397 (7)	0.0216 (3)	

### Atomic displacement parameters (Å^2^)

**Table:**

	*U*^11^	*U*^22^	*U*^33^	*U*^12^	*U*^13^	*U*^23^
C1	0.0173 (6)	0.0207 (6)	0.0253 (7)	0.0017 (5)	−0.0029 (5)	−0.0020 (5)
C11	0.0196 (6)	0.0224 (7)	0.0270 (7)	−0.0032 (5)	0.0008 (5)	−0.0018 (6)
C111	0.0374 (8)	0.0291 (8)	0.0297 (8)	−0.0099 (6)	−0.0043 (6)	−0.0038 (6)
C112	0.0272 (7)	0.0230 (7)	0.0360 (8)	−0.0018 (6)	−0.0002 (6)	−0.0029 (6)
C2	0.0211 (6)	0.0224 (7)	0.0232 (6)	0.0006 (5)	−0.0012 (5)	−0.0023 (6)
C3	0.0198 (6)	0.0203 (7)	0.0261 (7)	0.0007 (5)	−0.0028 (5)	0.0000 (5)
C31	0.0275 (6)	0.0212 (7)	0.0261 (7)	−0.0054 (5)	−0.0002 (5)	−0.0001 (6)
C311	0.0401 (8)	0.0279 (8)	0.0317 (8)	0.0006 (6)	−0.0002 (6)	0.0036 (6)
C312	0.0277 (7)	0.0324 (8)	0.0342 (8)	−0.0036 (6)	0.0050 (6)	−0.0010 (7)
C4	0.0204 (6)	0.0191 (6)	0.0280 (7)	−0.0004 (5)	−0.0041 (5)	−0.0030 (5)
C5	0.0250 (6)	0.0243 (7)	0.0280 (7)	0.0024 (5)	−0.0045 (5)	−0.0052 (6)
C6	0.0276 (7)	0.0324 (8)	0.0248 (7)	0.0061 (6)	−0.0003 (5)	−0.0050 (6)
C7	0.0230 (6)	0.0314 (8)	0.0279 (7)	0.0023 (6)	0.0032 (5)	0.0015 (6)
C8	0.0193 (6)	0.0257 (7)	0.0285 (7)	−0.0005 (5)	−0.0005 (5)	−0.0017 (6)
C9	0.0164 (6)	0.0212 (6)	0.0257 (7)	0.0035 (5)	−0.0020 (5)	−0.0012 (5)
C10	0.0182 (6)	0.0211 (7)	0.0253 (7)	0.0042 (5)	−0.0035 (5)	−0.0024 (5)

### Geometric parameters (Å, °)

**Table:**

C1—C2	1.3738 (18)	C311—H31A	0.9800
C1—C9	1.4400 (18)	C311—H31B	0.9800
C1—C11	1.5256 (18)	C311—H31C	0.9800
C11—C111	1.5291 (19)	C312—H31D	0.9800
C11—C112	1.5344 (19)	C312—H31E	0.9800
C11—H11	1.0000	C312—H31F	0.9800
C111—H11A	0.9800	C4—C10	1.4199 (18)
C111—H11B	0.9800	C4—H4	0.9500
C111—H11C	0.9800	C5—C6	1.369 (2)
C112—H11D	0.9800	C5—C10	1.4203 (18)
C112—H11E	0.9800	C5—H5	0.9500
C112—H11F	0.9800	C6—C7	1.407 (2)
C2—C3	1.4228 (17)	C6—H6	0.9500
C2—H2	0.9500	C7—C8	1.3701 (19)
C3—C4	1.3680 (18)	C7—H7	0.9500
C3—C31	1.5193 (18)	C8—C9	1.4236 (18)
C31—C311	1.5299 (19)	C8—H8	0.9500
C31—C312	1.5318 (19)	C9—C10	1.4273 (18)
C31—H31	1.0000		
			
C2—C1—C9	118.42 (12)	C31—C311—H31A	109.5
C2—C1—C11	121.44 (12)	C31—C311—H31B	109.5
C9—C1—C11	120.05 (11)	H31A—C311—H31B	109.5
C1—C11—C111	114.07 (11)	C31—C311—H31C	109.5
C1—C11—C112	110.04 (10)	H31A—C311—H31C	109.5
C111—C11—C112	109.70 (11)	H31B—C311—H31C	109.5
C1—C11—H11	107.6	C31—C312—H31D	109.5
C111—C11—H11	107.6	C31—C312—H31E	109.5
C112—C11—H11	107.6	H31D—C312—H31E	109.5
C11—C111—H11A	109.5	C31—C312—H31F	109.5
C11—C111—H11B	109.5	H31D—C312—H31F	109.5
H11A—C111—H11B	109.5	H31E—C312—H31F	109.5
C11—C111—H11C	109.5	C3—C4—C10	121.29 (12)
H11A—C111—H11C	109.5	C3—C4—H4	119.4
H11B—C111—H11C	109.5	C10—C4—H4	119.4
C11—C112—H11D	109.5	C6—C5—C10	121.09 (13)
C11—C112—H11E	109.5	C6—C5—H5	119.5
H11D—C112—H11E	109.5	C10—C5—H5	119.5
C11—C112—H11F	109.5	C5—C6—C7	119.93 (13)
H11D—C112—H11F	109.5	C5—C6—H6	120.0
H11E—C112—H11F	109.5	C7—C6—H6	120.0
C1—C2—C3	123.20 (12)	C8—C7—C6	120.54 (13)
C1—C2—H2	118.4	C8—C7—H7	119.7
C3—C2—H2	118.4	C6—C7—H7	119.7
C4—C3—C2	118.45 (12)	C7—C8—C9	121.34 (13)
C4—C3—C31	121.27 (12)	C7—C8—H8	119.3
C2—C3—C31	120.26 (12)	C9—C8—H8	119.3
C3—C31—C311	111.68 (11)	C8—C9—C10	117.81 (12)
C3—C31—C312	111.07 (11)	C8—C9—C1	123.32 (12)
C311—C31—C312	110.93 (12)	C10—C9—C1	118.87 (11)
C3—C31—H31	107.7	C4—C10—C5	121.02 (12)
C311—C31—H31	107.7	C4—C10—C9	119.74 (12)
C312—C31—H31	107.7	C5—C10—C9	119.24 (12)
			
C2—C1—C11—C111	−23.24 (17)	C6—C7—C8—C9	−0.8 (2)
C9—C1—C11—C111	160.20 (12)	C7—C8—C9—C10	−1.32 (18)
C2—C1—C11—C112	100.54 (14)	C7—C8—C9—C1	178.96 (12)
C9—C1—C11—C112	−76.03 (14)	C2—C1—C9—C8	178.25 (12)
C9—C1—C2—C3	0.37 (18)	C11—C1—C9—C8	−5.08 (18)
C11—C1—C2—C3	−176.25 (11)	C2—C1—C9—C10	−1.47 (17)
C1—C2—C3—C4	0.52 (19)	C11—C1—C9—C10	175.20 (11)
C1—C2—C3—C31	179.01 (12)	C3—C4—C10—C5	178.59 (12)
C4—C3—C31—C311	−117.29 (14)	C3—C4—C10—C9	−0.82 (18)
C2—C3—C31—C311	64.27 (16)	C6—C5—C10—C4	178.83 (12)
C4—C3—C31—C312	118.31 (14)	C6—C5—C10—C9	−1.76 (19)
C2—C3—C31—C312	−60.14 (15)	C8—C9—C10—C4	−178.04 (11)
C2—C3—C4—C10	−0.29 (18)	C1—C9—C10—C4	1.70 (17)
C31—C3—C4—C10	−178.76 (11)	C8—C9—C10—C5	2.54 (17)
C10—C5—C6—C7	−0.3 (2)	C1—C9—C10—C5	−177.73 (11)
C5—C6—C7—C8	1.6 (2)		

### Hydrogen-bond geometry (Å, °)

**Table:**

Cg1 and Cg2 are the centroids of the C1–C4/C9/C10 and C5–C10 rings, respectively.

**Table:**

*D*—H···*A*	*D*—H	H···*A*	*D*···*A*	*D*—H···*A*
C8—H8···Cg2^i^	0.95	2.88	3.7399 (15)	151
C11—H11···Cg1^i^	1.00	2.98	3.8289 (13)	144
C31—H31···Cg2^ii^	1.00	2.68	3.6048 (14)	155

Symmetry codes: (i) *x*, −*y*−3/2, *z*−1/2; (ii) *x*+3/2, −*y*+1/2, −*z*.
